# Connectivity Among Populations of the Top Shell *Gibbula divaricata* in the Adriatic Sea

**DOI:** 10.3389/fgene.2019.00177

**Published:** 2019-03-08

**Authors:** Violeta López-Márquez, José Templado, David Buckley, Ilaria Marino, Elisa Boscari, Dragos Micu, Lorenzo Zane, Annie Machordom

**Affiliations:** ^1^Museo Nacional de Ciencias Naturales – Consejo Superior de Investigaciones Científicas, Madrid, Spain; ^2^Centre D’Estudis Avançats de Blanes – Consejo Superior de Investigaciones Científicas, Girona, Spain; ^3^Departamento de Biología (Unidad de Genética), Universidad Autónoma de Madrid, Madrid, Spain; ^4^Department of Biology, University of Padova, Padova, Italy; ^5^National Institute for Marine Research and Development “Grigore Antipa”, Constanta, Romania; ^6^Consorzio Nazionale Interuniversitario per le Scienze del Mare, Rome, Italy

**Keywords:** Adriatic Sea, genetic connectivity, *Gibbula divaricata*, microsatellites, dispersal capacity

## Abstract

Genetic connectivity studies are essential to understand species diversity and genetic structure and to assess the role of potential factors affecting connectivity, thus enabling sound management and conservation strategies. Here, we analyzed the patterns of genetic variability in the marine snail *Gibbula divaricata* from five coastal locations in the central-south Adriatic Sea (central Mediterranean) and one in the adjacent northern Ionian Sea, using 21 described polymorphic microsatellite loci. Observed and expected heterozygosity varied from 0.582 to 0.635 and 0.684 to 0.780, respectively. AMOVA analyses showed that 97% of genetic variation was observed within populations. Nevertheless, significant, although small, genetic differentiation was found among nearly all of the pairwise *F*_ST_ comparisons. Over a general pattern of panmixia, three groups of populations were identified: eastern Adriatic populations, western Adriatic populations, and a third group represented by the single northern Ionian Sea population. Nonetheless, migration and gene flow were significant between these groups. *Gibbula divaricata* is thought to have a limited dispersal capacity related to its lecithotrophic trochophore larval stage. Our results indicated high levels of self-recruitment and gene flow that is mainly driven through coastline dispersion, with populations separated by the lack of suitable habitats or deep waters. This stepping-stone mode of dispersion together with the high levels of self-recruitment could lead to higher levels of population structuring and differentiation along the Adriatic Sea. Large effective population sizes and episodic events of long-distance dispersal might be responsible for the weak differentiation observed in the analyzed populations. In summary, the circulation system operating in this region creates natural barriers for dispersion that, together with life-history traits and habitat requirements, certainly affect connectivity in *G. divaricata*. However, this scenario of potential differentiation seems to be overridden by sporadic events of long-distance dispersal across barriers and large effective population sizes.

## Introduction

Connectivity among populations determines the dynamics of metapopulation systems, how genetic diversity arises and is maintained within species, and the adaptability and resilience of populations to environmental changes, among other factors (Botsford et al., 2001). Defining connectivity patterns for marine organisms, however, is a challenging task since factors that affect connectivity (e.g., life history traits, habitat, hydrological regime, occurrence of geological/topographical boundaries, layout of coastline, etc.) act at very different geographic and temporal scales (Villamor et al., 2014). For instance, processes related to dispersion, which ultimately determine patterns of connectivity, are highly linked to the biology and ecology of species, but they are also contingent on the evolutionary history of the group and the geological history of the inhabited area. More taxon-specific analyses are, therefore, needed to better understand how dispersion and connectivity of marine species are shaped through time and geographic space, and the evolutionary and ecological consequences they have on species.

In this study, we focus on *Gibbula divaricata*, a common species of shallow-water trochid gastropods (commonly known as top shells). This species was recently reassigned to the genus *Steromphala* Gray, 1847 (Affenzeller et al., 2017) due to previous descriptions of *Gibbula* as a paraphyletic group (Williams et al., 2010; Uribe et al., 2017). However, this reassignment lacks strong phylogenetic support indicating that more complete genetic and morphological analyses of the genus are still needed. Therefore, we have chosen to maintain the original genus name. *Gibbula divaricata* is distributed throughout the Mediterranean Sea (Templado, 2011) on shallow, sheltered rocky bottoms, including coastal lagoons, harbors and artificial hard structures. This species has also been cited along the coasts of the Black Sea (Anistratenko, 2005). It typically has a patchy pattern of distribution, with dense populations found in more favorable habitats but not in sandy beaches and rocky coastlines exposed to waves. Trochid gastropods produce lecithotrophic trochophore larvae that remain in the plankton for a short period (Hickman, 1992), usually less than 10 days (Underwood, 1972). *Gibbula divaricata* releases eggs into the water where fertilization takes place. Encapsulated larval development is fast: larvae hatch from the egg capsules 12 h post-fertilization and then remain in the plankton for a few days (Chukhchin, 1960). Although the exact duration of the planktotrophic larval stage of *G. divaricata* has not yet been determined, data from a closely related species, *Gibbula varia* (see Barco et al., 2013), indicate that development takes approximately 4 days from fertilization to complete metamorphosis and settlement (Samadi and Steiner, 2010). Given its distribution and presumptive limited dispersal capacity, *G. divaricata* represents an ideal organism to study genetic patterns at various geographic scales, and the potential historical and contemporary factors that have led to current biogeographic and genetic patterns.

This study focuses on central and southern populations of *G. divaricata* in the Adriatic basin. This basin represents a distinct marine sub-region with a priority status for marine spatial planning. This region is characterized by the existence of three cyclonic gyres (the north, the central and the southern Adriatic sub-gyres) and high differences on the bathymetry, temperature, chlorophyll-like pigment concentration, among others factors that differentiate up to six different regions (Barale et al., 2005). These factors have probably influenced the existence of biogeographical regions characterized by different categories of marine biota (Bianchi and Morri, 2000). However, some studies showed that these potential biogeographic boundaries cannot be generalized across species (Villamor et al., 2014).

The establishment of a large transboundary marine protected area has been proposed for the region (Bastari et al., 2016). The central-south sampling area was established by a pilot study within the European project COCONET (EU Seventh Framework Programme), which aims to acquire broad knowledge of marine protected areas using various approaches. Consequently, our sampling of *G. divaricata* was mainly confined to this predefined area. Genetic studies of other species in this area that have larval stages of varying durations have shown differing patterns of genetic differentiation, such as the fishes *Mullus barbatus* (Garoia et al., 2004), *Diplodus sargus* (Di Franco et al., 2012; Pujolar et al., 2013), *Tripterygion delaisi* (Koblmüller et al., 2015), *Scorpaena porcus* (Boissin et al., 2016) and *Symphodus tinca* (Carreras et al., 2017), the green crabs *Carcinus maenas* and *C. aestuarii* (Marino et al., 2011; Schiavina et al., 2014), the marble crab *Pachygrapsus marmoratus* (Fratini et al., 2016), the sea urchin *Paracentrotus lividus* (Paterno et al., 2017), and the seagrass *Posidonia oceanica* (Jahnke et al., 2017). These studies provide a good framework to compare and test the homogeneity or heterogeneity of dispersion and connectivity patterns within the same region.

The biology and dispersal capacity of *G. divaricata*, along with the distinctive physical features of the Adriatic Sea, provide a complex scenario in which to study population connectivity. Furthermore, small-scale processes are known to be important in generating patterns in benthic assemblages, supporting the idea that small-scale spatial variance contribute to regional- and broad-scale patterns of variation (Fraschetti et al., 2005).

In this study, we used microsatellite markers previously developed for *G. divaricata* (López-Márquez et al., 2016) to analyze central and southern Adriatic populations of the species in order to identify patterns in population structure, estimate connectivity at regional and local scales, and detect putative barriers to dispersion. Our analyses of genetic connectivity provide insight on the patterns of genetic structuring of *G. divaricata* in the central-south Adriatic Sea and point to contemporary factors (e.g., life history, habitat, currents, topography) potentially involved in such structuring. Genetic connectivity studies also provide valuable information for conservation and management plans in the area. For instance, coastal erosion and subsidence, among other factors such as the positive eustatism of the sea, will continue to affect the coastline for years to come (Brambati, 1992). These changes could result in the loss of suitable habitats for the species, and in the establishment of new barriers to dispersal and biogeographic boundaries along the Adriatic coastline, all of which can be highlighted by studying connectivity.

## Materials and Methods

### Study Area

**FIGURE 1:**
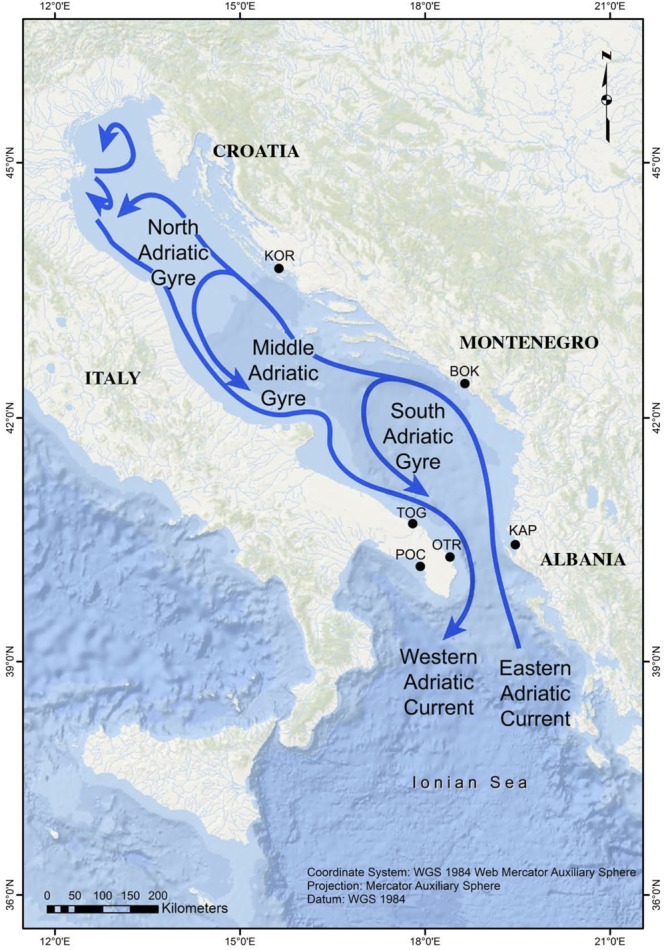
Map showing the sampling locations in the Adriatic Sea and its main surface currents (adapted from Melià et al., 2016).

The Adriatic Sea (Figure 1) is the most continental basin of the Mediterranean Sea (excluding the Black Sea) and is connected to the Ionian Sea through the Otranto Channel, which is 74 km wide. This latitudinally elongated sea is about 800 km in length (in its major axis, oriented from SE to NW) and has a mean width of 180 km. The Adriatic Sea is divided into three sectors: (1) the northern sector is shallow (average depth = 40 m) and has a gentle slope, (2) the middle sector (average depth = 140 m) has two depressions that reach depths of up to 260 m, and (3) the southern sector is where a wide depression is found (1,200 m deep) (Artegiani et al., 1996).

The predefined study area is comprised of eight localities, most of which are located in the southern sector. However, no *G. divaricata* specimens were found on Tremiti (42° 8′ 4.23″N; 15°31′38.59″E) or Othonoi (39° 50′ 29.60″N; 19° 24′ 8.92″E) islands, which, at least in the latter case, is likely due to the presence of *Gibbula rarilineata*, and its occupation of suitable habitats. The six remaining localities (Table 1 and Figure 1) are separated by distances ranging from 73 to 590 km (crossing the sea). Three locations along the eastern Adriatic coast (Karaburun Peninsula in Albania = KAP, Boka Kotorska in Montenegro = BOK, and Murter in Kornati National Park in Croatia = KOR) are up to 360 km apart. The other three localities are located along the Apulian coast of Italy, with two, separated by about 90 km, in the Adriatic side (Torre Guaceto = TOG and Otranto = OTR) and the third in the Gulf of Taranto (Porto Cesareo = POC) in the northern Ionian Sea. The localities OTR and POC are separated from each other by about 120 km of coastline, circumventing the end of the Apulian Peninsula. The localities KAP and OTR occupy opposite sides of the south Adriatic Sea (73 km apart crossing the sea) across the Otranto Sill.

**Table 1:** Location of *Gibbula divaricata* samples.

Location name	Population label	GPS coordinates	Sample size
Karaburun Peninsula (Valona, Albania)	KAP	40° 26′ 35″N; 19° 29′ 08″E	16
Boka Kotorska (Montenegro)	BOK	42° 24′ 888″N; 18° 38′ 111″E	35
Murter, Kornati National Park (Croatia)	KOR	43° 46′ 31″N; 15° 37′ 51″E	30
Torre Guaceto (Italy)	TOG	40° 42′ 999″N; 17° 48′ 003″E	30
San Foca, Otranto (Italy)	OTR	40° 18′ 12″N; 18° 24′ 17″E	29
Porto Cesareo (Italy)	POC	40° 11′ 715″N; 17° 55′ 077″E	34

The coast of Apulia extends for several hundred kilometers and is characterized by long and regular stretches of calcarenitic rock, divided by sandy beaches, with a gentle slope. It is also exposed to W–NW winds. The eastern Adriatic coast is irregular, with a series of large and small islands and a bathymetric profile comprised of rocky, steeply sloping areas, which can result in abrupt changes in habitat types.

The general surface circulation in the Adriatic Sea is cyclonic with a flow to the northwest along the eastern side and coming back to the southeast along the western side (Russo and Artegiani, 1996). Ionian waters enter on the eastern flank of the Otranto Channel and flow north-westward off Albania and Croatia by the Eastern Adriatic Current (EAC) (Poulain and Hariri, 2013). The EAC current re-circulates along the Italian coast, forming the permanent South Adriatic Gyre, which is the principal site where the Adriatic Deep Water forms. The Western Adriatic Current (WAC) is a strong coastal current that flows toward the southeast, with its surface waters eventually exiting through the western side of the Otranto Channel. Intra basin-scale circulation is dominated by seasonal cyclonic gyres of variable intensity, with waters from the EAC splitting and crossing the basin (Figure 1). The sub-gyre of the southern Adriatic tends to persist year-round (Russo and Artegiani, 1996). In conclusion, the mean cyclonic circulation favors alongshore connections and westward cross-Adriatic transport via the northern arms of the central and southern Adriatic sub-gyres (Carlson et al., 2016). This oceanographic pattern, which divides the area into three sub-regions that are connected to a larger circulation system and a series of temporal connections, greatly influences seascape connectivity within the basin (Melià et al., 2016). On the basis of large-scale connectivity patterns observed using different metrics, Dubois et al. (2016) noted that the Adriatic Sea has a persistent larval sink along the Puglia coast and the Gulf of Taranto, meaning that this area depends on other (source) populations to maintain its own populations.

### Sample Collection

Between 16 and 35 specimens of *G. divaricata* were collected from each locality (Table 1). Live specimens (kept alive on ice) were photographed prior to fixation with ethanol. As the operculum of trochid gastropods can sometimes prevent thorough fixation of the inner tissues, shells were first cracked with a small chisel or a vice clamp. The mollusc tissues were then removed and preserved in vials with absolute ethanol, and the shells were conserved for further morphological identification. The samples were stored at 4°C prior to DNA extraction. Intact shells, if available, were deposited into the Malacological Collection at the Museo Nacional de Ciencias Naturales in Madrid (MNCN 15.05/80173).

### DNA Extraction, Microsatellite Amplification, and Genotyping

Genomic DNA was extracted from a small piece of foot tissue. DNA was purified using the QIAGEN BioSprint 15 DNA Blood Kit (Qiagen), according to the manufacturer’s protocol, including a RNase treatment. DNA was quantified with the Quant-iT dsDNA HS Assay using a Qubit fluorometer, according to the manufacturer’s instructions. Aliquots of 2 ng/μl were prepared for subsequent genotyping analyses. DNA quality was also checked on 1% agarose gels. All individuals were identified to the species level by molecular and (when possible) morphological determination. Molecular identification was made by DNA barcoding following Barco et al. (2013). A 658 bp fragment at the 5′ end of cytochrome c oxidase subunit I (COI) was amplified using the primers LCO1491 (Folmer et al., 1994) and COI-H (Machordom et al., 2003). Sequences were compared with those available in GenBank using the BLAST algorithm (Altschul et al., 1997).

Microsatellites specific for *G. divaricata* (López-Márquez et al., 2016) were initially tested using nested-PCR conditions following the protocol developed for the mollusc *Panopea abbreviata* and the nemertean *Malacobdella arrokeana* (Ahanchédé et al., 2013; Alfaya et al., 2014, respectively). This consisted in a two steps PCR: in the first round, a forward primer with a tail at its 5′ end (PaulAn, see Acevedo et al., 2009) and a reverse primer were used. The product of this first reaction was used as template for the second, where the primers were PaulAn fluorescently 5′ end labeled with 6-FAM, NED, PET or VIC, and the reverse.

To amplify all populations, a three-primer PCR procedure (Vartia et al., 2014) was used. That included the forward primer with the tail at its 5′ end, the reverse primer, and the fluorescently 5′ end labeled tail. Alleles sizes were determined relative to the ABI GS500 LIZ standard. Based on electropherogram patterns and polymorphisms, 23 of the 26 loci described by López-Márquez et al. (2016) were selected for further analysis.

### Data Analysis and Genetic Diversity

We used MICRO-CHECKER v2.2.3 (Van Oosterhout et al., 2004) to test for the presence of null alleles (with a 95% confidence interval) and scoring errors. When the estimated frequency of the putative null allele was higher than 5% and/or the heterozygosity deficit was not corrected after considering the adjusted genotypes, the involved loci were eliminated.

Allelic richness was estimated for a standardized sample of 16 individuals per population using the R package STANDARICH (Alberto, 2006). Allelic diversity (Na), observed (Ho) and expected (He) heterozygosities, Hardy–Weinberg equilibrium (HWE) within populations for each locus and linkage disequilibrium were calculated using GENEPOP v4.0 (Raymond and Rousset, 1995) and GenAlEx 6.0 (Peakal and Smouse, 2006). In the statistical analyses, *p*-values were adjusted with Benjamini and Hochberg’s false discovery rate (Benjamini and Hochberg, 1995; Benjamini and Yekutieli, 2001).

Inbreeding coefficient values (*F*_IS_) were estimated for each population with GenAlEx. We also tested the influence of null alleles and genotyping failures on *F*_IS_ with INEST 2.2 (Chybicki and Burczyk, 2009). Inbreeding coefficients (for each population and the mean for the six populations pooled together) and the limit of the highest density posterior interval were calculated. To detect the existence of inbreeding effects in our data, a Bayesian approach using two different models (individual inbreeding models) was performed (with 50,000 burn-in cycles; 500,000 cycles overall; and 250 retained at each update). The first model, nbf, considered null alleles, inbreeding coefficients, and genotyping failures; the second model, nb, did not consider the inbreeding coefficients. To estimate the best-fit model, the deviance information criterion (DIC) was used.

Genetic relatedness was estimated by calculating pairwise relatedness in GenAlEx, considering all samples as a single population. Lynch and Ritland (1999) estimator and mean values were also assessed.

Finally, we estimated effective population sizes (Ne) for the six populations, using linkage disequilibrium, heterozygosity excess, and molecular co-ancestry methods, as implemented in NeEstimatorv2.1 (Do et al., 2014). We estimated the Ne values together with their confidence intervals (CIs) either by a parametric chi-square approximation or by jackknifing over individuals.

### Population Differentiation

Data from the 23 microsatellite loci genotyped were used to estimate genetic differentiation within and among populations. We calculated Wright’s fixation indices (*F*_ST_) using Weir and Cockerham’s estimators with GENETIX v.4.03 (Belkhir et al., 2004). Standardized *F*_ST_ values were also obtained by recoding the data matrix, assuming different alleles in each population for each locus, while maintaining their observed allelic frequencies. The standardized *F*_ST_ was calculated by dividing the original *F*_ST_ value by the corresponding recoded one. Hedrick (2005) and Meirmans and Hedrick (2011) used this approach for G_ST_ estimators and indicated that it could be used for *F*_ST_ indices to correct the bias of *F*_ST_ dependency on within-population diversity. Principal coordinates analyses (PCoA) were performed in GenAlEx with the obtained *F*_ST_ values. Furthermore, we used the function *betas* β in R with the Hierfstat package to calculate population-specific *F*_ST_ values (Weir and Hill, 2002).

We searched for *F*_ST_ outliers with the software LOSITAN (Antao et al., 2008), testing each locus for deviations from neutral expectations of the relationship between heterozygosity and *F*_ST_. Positive selection was inferred from the analysis if the given *p*-value was greater than 0.95. A Bayesian approach using BAYESCAN 2.01 (Foll, 2012) was performed with the following parameters: burn-in = 50,000, thinning interval = 30, number of outputted iterations = 5,000, number of pilot runs = 50, and length of pilot runs = 5,000. An R function called “plot_bayescan” was also run to plot and identify outliers. To compare the probability of a *p*-value, a Bayes factor was used, which provides a scale of evidence in favor of a selection model versus a neutral model following Jeffreys’s (1961) scale of evidence. We used STRUCTURE 2.2.3 (Pritchard et al., 2000) to infer the number of genetically differentiated populations (*K*) with the highest posterior probability. An admixture model was used under both the “popinfo” and the “popinfo” plus “location prior” functions with correlated allele frequencies. Twenty replicates per *K* were performed for *K* = 1–10 to calculate the mean log probability of the data [lnP(*K*)]. We exceed the number of analyzed populations to explore the potential existence of individuals belonging to locations outside the study area. MCMC iterations were set to 10,000 burn-in iterations and 100,000 sampled iterations. STRUCTURE Harvester (Earl and vonHoldt, 2012) was used to calculate Δ*K* values using the method proposed by Evanno et al. (2005). ARLEQUIN v3.5 (Excoffier et al., 2005) was then used to hierarchically quantify the molecular variance (AMOVA, *n* = 10,000 permutations) in the groups inferred by STRUCTURE. Clumpak (Kopelman et al., 2015) was used to compare the different results across the 20 replicates per *K*. A pattern of isolation by distance, through the correlation between pairwise multilocus differentiation [*F*_ST_/(1 -*F*_ST_)] and geographical distances (Ln distance), was assessed using the Mantel permutation test (10,000 permutations; Mantel, 1967) implemented in GenAlEx. Two methods were used to calculate geographical distances in kilometers: (1) Euclidean (straight line) distance (across the sea) between sampled locations and (2) the coastline distance between sampled locations.

Potential gene flow barriers were identified using BARRIER v.2.2 (Manni et al., 2004). Putative barriers were computed on a Delaunay triangulation, built with GPS coordinates, using Monmonier (1973) maximum difference algorithm with the pairwise *F*_ST_ matrix. The robustness of the identified barriers was tested with 100 resampled bootstrap matrices created with a R function (provided by Eric Petit, UMR ECOBIO CNRS).

We also investigated patterns of migration and individual ancestries among populations using Bayesian and coalescence tools. The three approaches used estimate migration at different time scales (among other parameters): Migrate-n estimates long-term or historical migration, BayesAss estimates recent migration, and GENCLASS2 identifies first generation migrants. For the first approach, implemented in Migrate-n 3.6 (Beerli and Palczewski, 2010), we ran some initial analyses with the six populations and an unrestricted pattern of migration between them to check for convergence and the adequacy of the priors for the mutation-scaled migration rates (*M*) and mutation-scaled effective population sizes (theta). We then ran a full analysis under a Brownian motion microsatellite model, generating the initial theta and *M* values using the default *F*_ST_ calculations, and uniform priors of 0–50 and 0–2000 for the two parameters, respectively. We ran one long MCMC chain with 10^5^ steps recorded and an increment of 100, excluding 10^4^ steps as burn-in. We also checked for the adequacy of this full population and migration model by comparing its probability (marginal likelihood) with that of three other demographic scenarios: panmixia [a single population (KAP+BOK+KOR+TOG+OTR+POC)], two populations [Adriatic (KAP+BOK+KOR) *vs.* Apulian (TOG+OTR) + Ionian (POC) sides], and three populations [Adriatic (KAP+BOK+KOR) *vs.* Apulian (TOG+OTR) *vs.* Ionian (POC) sides]. To estimate the marginal likelihood for each of these four scenarios, we ran new analyses with the same priors as above and four MCMC chains with a static heating scheme (temperatures: 1, 1.5, 3, 10^6^). We also ran two analyses with asymmetrical models of gene flow, the first one favoring counter-clockwise dispersal, following the sea surface currents in the Adriatic, and the second one that goes against sea surface currents. These analyses were run in the Cipres Science Gateway v3.3 (Miller et al., 2010). We then compared the marginal likelihood (Bezier approximation score) of the six models and ranked them according to their probability calculated with log Bayes factors in mtraceR (Pacioni et al., 2015).

We ran a BayesAss analysis to estimate recent migration rates (*m*) between populations with the following parameters: number of iterations, 10,000,000; sampling frequency, 100; length of burn-in 1,000,000; delta allele, 0.1; delta migration, 0.2; and delta *F*, 0.1. Probabilities of exclusion and inclusion were also calculated. If the probability of exclusion was greater than 95%, an individual was excluded from its sampling site; it was re-assigned to one of the other sampled populations if the probability of inclusion was greater than 10% (Underwood et al., 2007). An individual was assumed to have originated from an unknown population if it was excluded from one population but not re-assigned to one of the others.

Finally, a Bayesian assignment method (Rannala and Mountain, 1997), implemented in GENECLASS2 (Piry et al., 2004), was performed to identify putative first-generation migrants among populations. Then, to compute individual probabilities of assignment to each population, a Monte Carlo resampling method (simulation algorithm Paetkau et al., 2004) was performed using 10,000 simulated individuals with a type I error of 0.05.

## Results

### Genetic Variability

The 23 loci analyzed were polymorphic for all populations. Standardized allelic richness over loci for each population ranged from 7.59 for OTR to 9.60 for POC (Table 2). The mean value over loci and populations was 8.64. Observed and expected heterozygosities ranged from 0.582 for TOG to 0.635 for BOK and from 0.684 for KAP to 0.780 for POC, respectively (with mean values of 0.6 and 0.722, respectively) (Table 2). All *F*_IS_ values were positive and high (0.144–0.253), indicating a heterozygosity deficit. When correcting the effect of null alleles on this parameter, the values decreased (avg. Fi = 0.016–0.078), and all highest posterior density intervals (HPDI = 0–0.143) included the zero value. Almost all populations showed nb as the best-fit model, thus reducing the role of inbreeding in the differences between observed and expected heterozygosities. For BOK and KOR, nfb was the best-fit model, although differences in DIC values between the two models were less than 1 (Table 2).

**Table 2:** Estimators of genetic diversity in 174 samples of *Gibbula divaricata* at 21 microsatellite loci.

Population	Na	Ho	He	*F*_IS_	Avg. Fi (95% HPDI)	DIC (nfb)	DIC (nb)	Pa (frequencies range)
KAP	8.476	0.589	0.684	0.171	0.025 (0–0.077)	2102.118	**2100.950**	0.381 (0.031–0.063)
BOK	9.235	0.635	0.731	0.144	0.016 (0–0.049)	**5083.644**	5083.833	1.524 (0.014–0.045)
KOR	9.065	0.620	0.713	0.147	0.078 (0–0.143)	**4270.947**	4271.897	1.238 (0.017–0.050)
TOG	7.917	0.582	0.702	0.183	0.016 (0–0.052)	3867.653	**3867.370**	0.667 (0.017–0.033)
OTR	7.598	0.585	0.719	0.198	0.038 (0–0.107)	3719.086	**3716.405**	0.571 (0.017–0.052)
POC	9.607	0.590	0.780	0.253	0.025 (0–0.077)	5204.090	**5202.547**	2.524 (0.015–0.103)
Mean	8.649	0.600	0.722	0.182	0.021 (0–0.054)	25,830.552	**25,828.967**	1.214

*Na, number of alleles; Ho, observed heterozygosity; He, expected heterozygosity; *F*_IS,_ inbreeding coefficient; Avg. Fi (95% HPDI), average of inbreeding coefficient estimated by INEST considering the null alleles (95% highest posterior density intervals): in bold, best model; DIC, deviance information criterion for nfb and nb models (n accounts for null alleles, f for inbreeding, and b for genotyping failures); Pa, mean number of private alleles, and in parentheses, the range of private allele frequencies.*

Linkage disequilibrium among loci was not observed in any of the populations, implying that the 23 loci can be considered statistically independent. Even after Benjamini and Hochberg corrections, all localities had significant deviations from HWE for different loci, usually due to heterozygote deficits, which may be accounted for by the presence of null alleles. Two loci (Gd-L22 and Gd-L42) presented high frequencies for all studied locations and were, thus, removed from subsequent analyses. Problems associated with amplification, scoring or mutations in the regions where primers were designed may account for the presence of null alleles at these two loci. Other reasons may account for the deviation from HWE for the other loci. For example, 31 alleles were found for the locus Gd-5 in KOR, where 30 specimens were analyzed. These 31 alleles can be combined to give 496 different genotypes [*n*(*n* + 1)/2]. However, in order to interpret the resulting HWE χ^2^ tests accurately, the sample size and the expected number for all genotypes should be more than 50 and 5, respectively (Hedrick, 2010). The correction made by the exact test could not resolve this problem. No statistically significant differences were observed in pairwise *F*_ST_ and pairwise *F*_ST_ corrected for null alleles. Given this, null alleles were not considered hereafter, thus avoiding the artificial creation of shared (null) alleles among populations.

Despite being considered neutral markers, LOSITAN identified two outlier loci: Gd-L3 and Gd-L32 showed significantly higher *F*_ST_ values than neutral expectations, indicating positive selection. BAYESCAN showed four loci (Gd-L5, Gd-L16, Gd-L37, and Gd-L38) as putative outliers under negative selection. Given the lack of agreement between the two tests of neutrality, all loci were considered for downstream analyses.

Estimates of pairwise relatedness among the samples ranged from -0.079 to 0.471; the mean value was -0.003. Plotting the indices, a unimodal distribution around zero was obtained (data not shown), indicating no relatedness among most specimens.

Effective population sizes for the six populations were, in general, very large (Supplementary Table S1). Either these Ne values were ‘infinite’ or the CIs included ‘infinite,’ meaning that the populations are large enough that genetic drift does not have a significant effect on the variation of the population genetic characteristics.

### Population Differentiation

The global value of *F*_ST_ revealed a low but significant level of genetic differentiation (*F*_ST_ global = 0.0246, *p* < 0.0001; standardized *F*_ST_ global = 0.0964). Pairwise *F*_ST_ values ranged from negative values (that should be considered as zero) for KOR *vs.* KAP and TOG *vs.* OTR to 0.0460 (0.1738 for the standardized *F*_ST_) for OTR *vs.* KAP (Table 3). Western Adriatic populations from the Apulian Peninsula (POC, OTR, and TOG) were significantly differentiated from eastern Adriatic populations (KAP, KOR, and BOK). The Ionian locality POC showed significant values with respect to the other localities. Population specific *F*_ST_ β ranged from -0.0469 for POC to 0.0661 for KAP (Supplementary Table S2).

**Table 3:** Genetic distances shown as *F*_ST_ (below the diagonal) and standardized *F*_ST_ values (above the diagonal).

	KAP	BOK	KOR	TOG	OTR	POC
KAP	0	0.00836	–0.00503	0.15048	0.16699	0.17380
BOK	0.00233	0	0.01312	0.10454	0.11308	0.15248
KOR	–0.00141	0.00347	0	0.09941	0.10751	0.14215
TOG	**0.04296**	**0.02817**	**0.02760**	0	–0.00273	0.08256
OTR	**0.04605**	**0.02951**	**0.02891**	–0.00075	0	0.09046
POC	**0.04195**	**0.03517**	**0.03380**	**0.02007**	**0.02120**	0

*Significant values (*P* < 0.01) for *F*_*ST*_ using Benjamini and Hochberg’s false discovery rate correction are indicated in bold.*

No significant association between genetic differentiation (*F*_ST_) and geographic distance was revealed when using Euclidean distances between locations across the sea (Mantel test, *P* = 0.440, *R* = 0.00091). In contrast, a higher correlation was found when geographic distances were measured following the coastline between sampled locations (Mantel test, *P* = 0.050, *R* = 0.62582).

**FIGURE 2:**
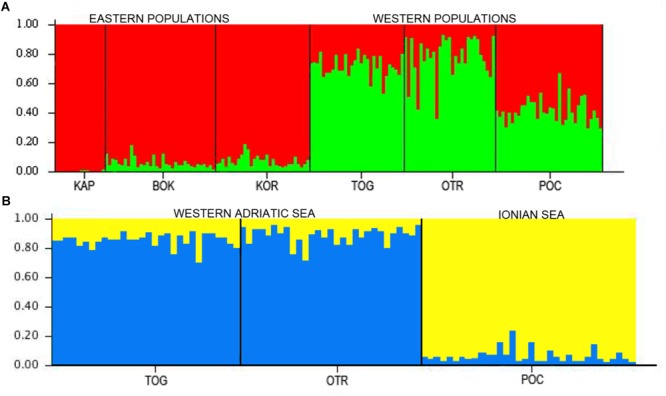
**(A)** STRUCTURE results for the six locations in the Adriatic and Ionian seas (*K* = 2). **(B)** STRUCTURE results for the three west locations in the Adriatic and Ionian seas (*K* = 2).

In the STRUCTURE analyses, two genetically differentiated clusters were detected under the functional parameters of both “popinfo” and “popinfo + location prior.” The highest value of likelihood was *K* = 2, supported by the highest Δ*K* values (Δ*K* = 17.77 and 22.28, respectively) (Supplementary Figure S1). The two clusters corresponded to populations belonging to the west (TOG, OTR, and POC) and east (KAP, BOK, and KOR) coasts of the Adriatic Sea, although most of the specimens from the western locations presented admixed ancestries (Figure 2A). Since the *F*_ST_ values indicated differentiation among the western populations, a second STRUCTURE analysis of this group was performed. POC was differentiated from the other western locations (TOG and OTR), a result that was supported by the highest Δ*K* value (Δ*K* = 7.80) (Figure 2B). In fact, POC has the greatest number of private alleles in almost all loci, some at frequencies around 10%.

AMOVA analysis of the eastern and western groups revealed that 96.64% of the genetic variation originated within populations and only 2.31% among populations. The lowest percentage of variation (1.05%) was among populations within the two groups (Table 4).

**Table 4:** AMOVA analyses for six populations of *G. divaricata* divided into two groups (east and west sides of Adriatic Sea).

Source of variation	d.f.	Sum of squares	Variance components	Percentage of variation
Among groups	1	43.012	0.177	2.31
Among populations within groups	4	48.028	0.080	1.05
Within populations	342	2540.781	7.429	96.64
Total	347	2631.822	7.687	

*P-values for all results were significant (*P* < 0.0001).*

**FIGURE 3:**
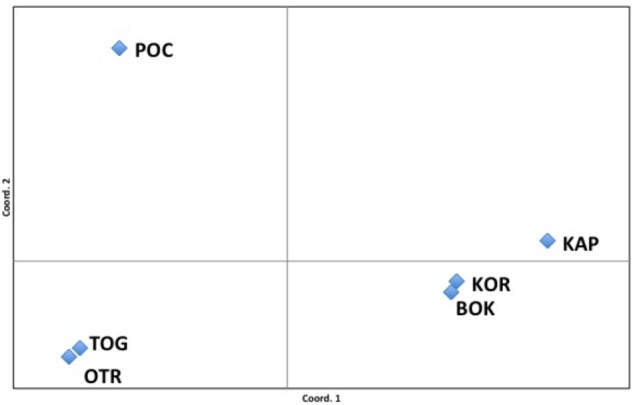
Principal coordinates analysis (PCoA). *F*_ST_ values among populations showed a variation of 96.81% explained by the two first axes.

The *F*_ST_ values among populations represented in the PCoA analysis showed that 96.81% of variation could be explained by the first two axes. The first axis of the PCoA clearly separated OTR, TOG and POC from the other Adriatic localities (Figure 3). Nevertheless, within this group, analysis of the second axis showed the well-supported division of POC from OTR and TOG.

Putative barriers of gene flow across the studied area were assessed with BARRIER. This analysis indicated a well-supported (bootstrap value = 96%) barrier between the east and the west coasts of the Adriatic Sea, dividing KAP, BOK, and KOR from TOG, OTR, and POC. The presence of a second barrier, separating POC from TOG and OTR, was also indicated but with lower support.

Migration rates among the six populations were estimated using a Bayesian coalescence approach (Migrate-n). The highest mutation-scaled migration rates (*M*) were generally found between populations on either the eastern or western sides of the Adriatic Sea [populations KAP, BOK, KOR or TOG, OTR, POC, respectively (Supplementary Table S3)], which seem to corroborate the results of the *F*_ST_, STRUCTURE, and Mantel test analyses. Migration rates from POC to the other five populations are, however, consistently high. Dispersal from TOG and OTR to the other populations (except to each other) seems to have been historically constrained, as evidenced by the low *M* estimated for these two populations. However, when the marginal likelihood of the full migration model among the six populations was compared with those of the other demographic and gene flow scenarios (panmixia, two populations, three populations, asymmetric counter-clockwise gene flow, and asymmetric clockwise gene flow), the results favored panmixia as the most likely scenario (Supplementary Table S4), followed by the two asymmetric gene flow scenarios, the latter mimicking a stepping-stone model of dispersion along the coastline.

In the BayesAss analysis, the highest migration rates (*m*) were found between populations from either the eastern or western sides (i.e., from BOK to KAP and KOR and from TOG to OTR and POC, respectively) (Supplementary Table S5). Recent emigration from OTR and POC, however, was minimal. GENECLASS2 detected 30 individuals as potential first generation migrants, corresponding to 17.2% of the total number of individuals analyzed (Supplementary Table S6). Remarkably, in this analysis, the largest number of putative first generation migrants originated from unknown populations, followed by the western population of POC (with most emigrating to OTR).

## Discussion

Connectivity among populations, and patterns of dispersal and gene flow, are primarily determined by the physical characteristics of the landscape occupied by a species and the biological life-history traits of that species. Connectivity and gene flow, in turn, shape the patterns of genetic structuring of a species, which are of profound ecological and evolutionary importance. In this study, we analyzed the genetic structure of the topshell *Gibbula divaricata* in the central and southern Adriatic Sea to infer its population structure and patterns of dispersal and gene flow. We found that, overall, populations seem well-connected and panmixia characterizes the species in the area. However, the presence of physical barriers to dispersal were also indicated in the area, and our results suggest that dispersion is mainly following the coastlines with help from the currents. One hypothesis is that this would eventually lead to high levels of genetic structuring and differentiation. However, this apparently is not the case in *G. divaricata*: we found evidence of weak though statistically significant differentiation among some of the populations, but overall panmixia is the rule. Large effective populations sizes and sporadic long-distance migration events among populations, as shown by the coalescence-based migration analyses, could explain this scenario. A large-scale study, nevertheless, would be necessary to estimate with more accuracy the patterns of gene flow, the demographic history of the species, and the precise physical environmental barriers influencing connectivity patterns in the species.

Overall, all of our analyses and tests consistently showed that connectivity was limited to some degree between the eastern and the western sites of the South Adriatic and, to a lesser extent, between Porto Cesareo in the Ionian Sea and the other two Italian localities along the Adriatic coast of the Apulian Peninsula. Taken together, our results indicate an Adriatic population (although panmictic) structured in two groups: one comprised of eastern populations and the other of western ones, although this last group showed a certain level of admixture (see Figure 2). Further analyses of the western populations revealed a third cluster, Porto Cesareo. A putative barrier between this location and the others was also found. This population showed the highest number of private alleles, some at frequencies near 10%, further supporting the presence of this third group. In any case, the barriers between eastern and western coasts of the Adriatic and between the Adriatic and Ionian coasts of Apulia are permeable as migrants were found among populations of each side (although most exchanges occurred between populations on the same coastline). Notably, the analyses of migration events indicate POC as a source population, both historically and currently. POC is the most differentiated population, and it is located at the edge of the Adriatic Sea. Therefore, the assigned migrants coming from POC (according to GENECLASS2) could be those considered as ‘unknown’ in the BayesAss analysis.

Genetic studies of the bentho-pelagic red mullet (*Mullus barbatus*) (Garoia et al., 2004) and the benthic black scorpionfish (*Scorpaena porcus*) (Boissin et al., 2016) have also revealed differences between the eastern and the western parts of the Adriatic basin. However, *G. divaricata* and *S. porcus* show differences in connectivity along the eastern Adriatic coast. Although we found no significant genetic differentiation of *G. divaricata* among our three sampled eastern populations, Boissin et al. (2016) showed strong differences among eastern populations of *S. porcus*, particularly between two samples from Croatia and Albania, highlighting how connectivity patterns cannot be generalized among species. A single biological trait (e.g., characteristics of the larval phase, self-recruitment or larval duration) can determine the capacity of larval exchange among populations and, hence, the extent and direction of dispersal (Melià et al., 2016). Likewise, synchronization between spawning and hydrodynamic conditions may also be a factor determining the extent of larval transport (Koeck et al., 2015).

We found no relationship between genetic and geographic distances when using Euclidean distances between locations; however, an association was found when geographic distances were measured following the coastline distances between sampled locations. Therefore, factors such as the current system, coastline layout, small-scale local hydrodynamic processes or favorable habitat availability are likely to have greater influence on the degree of connectivity between different local populations than distance itself.

Our data indicate that trochophore larvae of *G. divaricata*, although thought to have limited dispersal abilities, can eventually reach and settle in distant populations. The precise timing of larval development in *G. divaricata* is still unknown; however, other species of the genus take no longer than 10 days to reach complete metamorphosis (Underwood, 1972). Lagrangian models that simulate drifter trajectories in the Adriatic Sea support sporadic and unexpected migrations, which may account for some of our connectivity results as it only takes 5 days (consistent with the presumed duration of the larval stage of *G. divaricata*) for particles to drift between TOG and OTR or POC (Carlson et al., 2016). Migration between these sites and others (e.g., KOR and KAP) is highly unlikely following these models as it can take more than 40 days for particles to drift from TOG to KOR (Poulain, 2001; Bray et al., 2017), which is much longer than the pelagic larval period assumed for *G. divaricata*.

Migration from POC to the other populations and between populations on either side of the Adriatic Sea, however, were not consistent with predominant surface currents/biophysical models (Figure 1), although local gyres might explain these results. In a recent study of the connectivity patterns of a Mediterranean wrasse, *Symphodus ocellatus*, which also has a short larval phase of about 10 days (Macpherson and Raventos, 2006), and the biophysical models in the Adriatic, Melià et al. (2016) suggested the coexistence of a high local retention potential of propagules with very occasional intense movement across the sea basin. On the other hand, according to these same authors, communities were significantly more similar along a gradient of connectivity by ocean currents than with increasing distance in the region encompassing the southern Adriatic and Ionian seas. Likewise, in our analysis, more differences were found between physically closer populations (KAP and OTR) than between remote populations linked by these ocean currents (KAP and KOR).

Genetic analyses of a variety of species in the Adriatic Sea (Garoia et al., 2004; Di Franco et al., 2012; Pujolar et al., 2013; Schiavina et al., 2014; Boissin et al., 2016; Carreras et al., 2017) have indicated that realized dispersal (larvae that actually travel from one locality to another) is much less pronounced than potential dispersal (probability of larval transport from a source to destination location, as quantified by Lagrangian particle simulations) as evidenced by some degree of differentiation among sites for some species. Panmictic species may represent exceptions. For example, the marbled crab *Pachygrapsus marmoratus*, given its ubiquity and long pelagic larval duration, was shown to have high connectivity within the Adriatic and even more broadly throughout the Mediterranean (Fratini et al., 2016). The sea urchin *Paracentrotus lividus* showed a similar pattern, but in this case, connectivity was limited to the Adriatic and Ionian seas (Paterno et al., 2017). The shore crab *Carcinus aestuarii* (Schiavina et al., 2014), which is a highly dispersive species (in lagoons and estuaries), may also have limited connectivity in these seas as females release larvae in coastal waters after a brief migration toward the open sea (Mori et al., 1990). The specificity of this species to isolated habitats, along with their great dispersal capacity, implies that oceanographic conditions play an important role in larval dispersion. In such cases, the translocation of pre-competent larvae from nearshore to offshore and the shoreward return (by onshore advection) of competent larvae should be favored, replenishing the larval supply to this species’ specific shore habitats. In the case of *G. divaricata*, and in spite of its assumed low dispersal capacity, its specific habitat requirements (shallow sheltered rocky coast, including harbor areas), together with the coastal currents, likely favors a degree of dispersal along the coastline. The differentiation observed in the Ionian population may be due to, among other factors, the absence of sheltered habitats at intermediate points between OTR and POC. The southern area of the Apulian Peninsula consists of a high rocky coast exposed to intense wave action (Damiani et al., 1988) that may restrict connectivity in the species. Therefore, in species with restricted or specific environmental requirements, habitat patchiness may greatly influence population connectivity by affecting local larval production and recruitment success based on the proportion of favorable habitats (Pinsky et al., 2012; Anadón et al., 2013).

However, unlike *G. divaricata*, species such as *C. aestuarii* and *S. porcus* showed no significant genetic differences between Apulian localities in the Adriatic (Torre Guaceto and Otranto) and Ionian (Porto Cesareo) seas (Schiavina et al., 2014; Boissin et al., 2016, respectively). Likewise, the biophysical models that showed infrequent and weak connections linking the eastern and western sides of the Adriatic also showed moderately intense fluxes connecting the southernmost areas of Apulia with the eastern part of the Gulf of Taranto (Melià et al., 2016). These findings agree with other data obtained from surface drifter deployments and Lagrangian simulations performed in the same area to describe larval dispersal of the white seabream *Diplodus sargus* (Pujolar et al., 2013). Some authors have suggested a phylogeographical discontinuity of the Adriatic Sea given the genetic distinctiveness of the populations of several species inhabiting this basin (Patarnello et al., 2007; Maltagliati et al., 2010). Nevertheless, these genetic discontinuities (inferred from mitochondrial markers) seem to be more related to the effects of historical events, such as periods of very low sea level. Present-day coastal marine currents connecting the Adriatic and the Ionian seas favor larval exchange and contemporary gene flow, although these processes may be partially restricted in species with a supposed low dispersal capacity, such as *G. divaricata*.

Genetic homogeneity may be explained by a continuous and significant coastal marine current that connects different areas, thus promoting larval exchange. Surface drifter tracks and modeled particle trajectories indicate that the Adriatic circulation patterns sustain physical corridors as connection pathways within a large-scale functional cell of connectivity in the southern Adriatic (Boero et al., 2016). Likewise, several persistent larval sinks have been noted along the southern Italian shore (Bray et al., 2017). Though these studies are informative about Adriatic alongshore coastal currents and cyclonic gyres, they may not reflect actual larval pathways, which in hydrodynamic connectivity studies, are based on drifter trajectories. Rather, larval behaviors range from passively drifting to selectively using currents for dispersal. Marine larvae display a large repertoire of behaviors that can greatly affect dispersal patterns at different spatial scales, such as the ability to migrate vertically, swim horizontally, or undergo ontogenetic changes in behavior or those related to having distinctive sensory capabilities. These behaviors allow larvae to change their dispersal pattern by taking advantage of different oceanographic conditions (Pineda et al., 2007). Habitat selection (at small spatial scales) is determined by the differential responses of larvae to proximate environmental stimuli (Kingsford et al., 2002). Hence, the interaction between larval behavior and physical processes can influence dispersal patterns to either increase advection from the native population or enhance self-recruitment (Sponaugle et al., 2002).

In order to determine if connectivity is mediated through a continuous stretch of suitable habitats along the coastline, genetic analyses need to be combined with habitat mapping. Species with supposed low dispersal abilities, such as *G. divaricata*, may use patches of its specific habitat as stepping stones, thus allowing them to disperse over a wider range. As a result of stepping stone dispersion, ubiquitous shore species that have access to relatively continuous habitats would show high genetic connectivity, while species inhabiting patchy dispersed habitats, or isolated habitats such as estuaries and lagoons, would show low to no connectivity. This connectivity, in turn, can be modulated by the durations and behaviors of the early life stages of different species.

Overall, our findings indicate weak differentiation among the studied populations belonging to the western and the eastern coasts of the central-south Adriatic. Although significant, the extent of differentiation and the strength of barriers were low, and some level of migration was evident, which taken together suggest very recent isolation or a weak restriction to gene flow. Multiple physical and biological factors may be influencing this population structure. Therefore, much research is still needed to determine the extent to which these factors affect connectivity in this species, not only in the Adriatic region but throughout its distribution. In addition, knowledge of the life cycle phases of *G. divaricata*, and their ecological interactions, will shed light on the actual dispersal ability of the species and enlighten ongoing phylogeographic studies.

Connectivity is increasingly recognized as a key conservation objective because of its importance for species replenishment (Saenz-Agudelo et al., 2011). Changes in fertilization success, larval supply, and recruitment play a major role in connectivity and, therefore, in both the future population dynamics and the long-term viability of a species. Regrettably, habitat fragmentation and loss due to human activities are widely considered one of the strongest drivers of change at all levels of biodiversity, as well as in the structuring and functioning of marine coastal ecosystems (Templado, 2014). Populations become more isolated (due to loss of connectivity) and decrease in size and abundance due to habitat fragmentation and loss. Therefore, conservation measures should aim to guarantee the persistence and continuity of natural habitats. The establishment of a well-planned network of marine protected areas will also help to maintain connectivity among populations.

## Author Contributions

VL-M conceived the research, conducted field work and laboratory and data analyses, and wrote the manuscript. JT and AM conceived the research, were responsible for resource acquisition, conducted field work and wrote and provided comments on the manuscript. DB collaborated in data analyses and reviewed the manuscript. LZ conducted field work, collaborated in data analyses, and provided comments on the manuscript. IM, EB, and DM conducted field work and reviewed the manuscript. This research is part of VL-M Ph.D. thesis, co-directed by JT and AM.

## Conflict of Interest Statement

The authors declare that the research was conducted in the absence of any commercial or financial relationships that could be construed as a potential conflict of interest.
