# Supplementary material for: Connectivity Among Populations of the Top Shell Gibbula divaricata in the Adriatic Sea
Source: Front Genet. 2019 Mar 8;10:177. doi: 10.3389/fgene.2019.00177 (PMC6418013; doi:10.3389/fgene.2019.00177)
Supplement: Supplementary file 1 [file Table_1.pdf]

LINKAGE DISEQUILIBRIUM METHOD, Mating Model: Random
Lowest allele frequencies used: 0.0500 0.0200 0.0100

							Parametric CI		Jackknife CI		
**Samp Size**	**PCrit.**	**Weighted Mean**	**Ind. Alleles**	**r^2**	**Exp(r^2)**	**Ne**	**Low**	**High**	**Low**	**High**	**(Eff.df)**
16	0.05	15.3	4752	0.090059	0.079569	27.6	19.8	42.4	5.2	Infinite	109
	0.02	15.2	10368	0.082461	0.079525	103.2	57.2	416.5	15.9	Infinite	324
	0.01	15.2	10368	0.082461	0.079525	103.2	57.2	416.5	15.9	Infinite	324
35	0.05	33.9	4335	0.031907	0.032214	-1091.2	306.3	Infinite	233.4	Infinite	2856
	0.02	34	13472	0.032026	0.03217	-2310.6	523.4	Infinite	327.2	Infinite	6214
	0.01	33.9	25681	0.031709	0.032263	-604.1	161803.5	Infinite	1083.1	Infinite	10855
30	0.05	28.9	5782	0.038916	0.038136	393.5	135.9	Infinite	96	Infinite	2261
	0.02	28.6	11154	0.03849	0.038446	7051.9	285	Infinite	162.5	Infinite	3638
	0.01	28.9	22767	0.038382	0.038154	1352.4	325.1	Infinite	188.9	Infinite	6191
30	0.05	28.4	5270	0.037648	0.038548	-344.1	528.9	Infinite	232.6	Infinite	2422
	0.02	27.9	8319	0.038398	0.039069	-461	586.5	Infinite	226.2	Infinite	2994
	0.01	28.6	14536	0.037781	0.038313	-580.7	873.3	Infinite	471.1	Infinite	8205
29	0.05	28.1	5473	0.040161	0.039515	474.6	138.6	Infinite	80.4	Infinite	1440
	0.02	28.2	8088	0.040219	0.039441	394.1	148.8	Infinite	123.6	Infinite	4690
	0.01	28.3	12980	0.039196	0.039244	-6468.8	331.7	Infinite	313.8	Infinite	11704
34	0.05	31.9	5636	0.037776	0.034323	94.4	66.1	156.9	25.8	Infinite	210
	0.02	32.2	14024	0.035562	0.03402	214	137.4	458.1	64.6	Infinite	939
	0.01	32.1	26653	0.034214	0.034123	3655.7	488.4	Infinite	139.1	Infinite	1925

HETEROZYGOTE EXCESS METHOD
Lowest allele frequencies used: 0.0500 0.0200 0.0100

**Samp Size**	**PCrit.**	**Harmonic Mean**	**Ind. Alleles**	**D**	**Ne**	**Parametric CI for Ne**	
16	0.05	15.4	102	-0.19506	Infinite	Infinite	Infinite
	0.02	15.3	151	-0.12516	Infinite	Infinite	Infinite
	0.01	15.3	151	-0.12516	Infinite	Infinite	Infinite
35	0.05	34.4	97	-0.15966	Infinite	Infinite	Infinite
	0.02	34.4	171	-0.14304	Infinite	Infinite	Infinite
	0.01	34.4	236	-0.10674	Infinite	Infinite	Infinite
30	0.05	29.1	112	-0.17536	Infinite	Infinite	Infinite
	0.02	29.1	154	-0.17598	Infinite	Infinite	Infinite
	0.01	29.1	223	-0.12164	Infinite	Infinite	Infinite
30	0.05	29	108	-0.15785	Infinite	Infinite	Infinite
	0.02	29	132	-0.14511	Infinite	Infinite	Infinite
	0.01	29	179	-0.10714	Infinite	Infinite	Infinite
29	0.05	28.4	109	-0.20939	Infinite	Infinite	Infinite
	0.02	28.4	132	-0.20355	Infinite	Infinite	Infinite
	0.01	28.4	168	-0.15557	Infinite	Infinite	Infinite
34	0.05	32.9	108	-0.27587	Infinite	Infinite	Infinite
	0.02	32.9	175	-0.23143	Infinite	Infinite	Infinite
	0.01	32.9	241	-0.16694	Infinite	Infinite	Infinite

MOLECULAR COANCESTRY METHOD

**Samp Size**	**Harmonic Mean**	**f^1**	**Neb^**	**Jackknife CI for Neb^**	
16	15.3	0.02559	19.5	4	47.1
35	34.4	0.00949	52.7	1.3	194.5
30	29.1	-0.01435	Infinite	Infinite	Infinite
30	29	-0.05117	Infinite	Infinite	Infinite
29	28.4	0.00345	145.1	0.1	728.5
34	32.9	-0.0088	Infinite	Infinite	Infinite
