# Supplementary material for: Connectivity Among Populations of the Top Shell Gibbula divaricata in the Adriatic Sea
Source: Front Genet. 2019 Mar 8;10:177. doi: 10.3389/fgene.2019.00177 (PMC6418013; doi:10.3389/fgene.2019.00177)
Supplement: Supplementary file 2 [file Table_2.pdf]

	Beta	2.50%	97.50%
**KAP**	0.0661	0.0271	0.1187
**BOK**	0.0195	-0.0122	0.0576
**KOR**	0.0416	0.0072	0.0839
**TOG**	0.0555	0.0198	0.0983
**OTR**	0.0329	0.0012	0.0611
**POC**	-0.0469	-0.1145	0.0043
