# Supplementary material for: Connectivity Among Populations of the Top Shell Gibbula divaricata in the Adriatic Sea
Source: Front Genet. 2019 Mar 8;10:177. doi: 10.3389/fgene.2019.00177 (PMC6418013; doi:10.3389/fgene.2019.00177)
Supplement: Supplementary file 3 [file Table_3.pdf]

	KAP	BOK	KOR	TOG	OTR	POC
**KAP**	***	11.796	18.183	11.552	7.050	15.445
**BOK**	8.340	***	9.481	11.197	5.166	6.703
**KOR**	6.177	8.511	***	11.534	6.001	9.162
**TOG**	7.062	14.644	9.069	***	21.670	17.991
**OTR**	7.493	10.133	9.010	8.885	***	24.196
**POC**	5.635	6.423	7.155	5.211	5.652	***

Row= population of origin
For instance: M2->1=11.796 (immigrants FROM population 2 IN population1)
