# Supplementary material for: Connectivity Among Populations of the Top Shell Gibbula divaricata in the Adriatic Sea
Source: Front Genet. 2019 Mar 8;10:177. doi: 10.3389/fgene.2019.00177 (PMC6418013; doi:10.3389/fgene.2019.00177)
Supplement: Supplementary file 4 [file Table_4.pdf]

	lmL	LBF	mod.rank	mod.prob
**Panmixia**	-33090.43	0	1	1
**Two-pops**	-293818	-521455,14	4	0
**Three-pops**	-596203.46	-1126226,06	5	0
**Six-pops**	-1730353.9	-3394526,86	6	0
**Asymmetric_1**	-217652.17	-369123,48	2	0
**Asymmetric_2**	-238600.2	-411019,54	3	0
