# Supplementary material for: Connectivity Among Populations of the Top Shell Gibbula divaricata in the Adriatic Sea
Source: Front Genet. 2019 Mar 8;10:177. doi: 10.3389/fgene.2019.00177 (PMC6418013; doi:10.3389/fgene.2019.00177)
Supplement: Supplementary file 5 [file Table_5.pdf]

Population	Origin					
	KAP	BOK	KOR	TOG	OTR	POC
KAP	0.6818	0.2505	0.0153	0.0221	0.0154	0.0150
BOK	0.0082	0.9287	0.0083	0.0386	0.0081	0.0082
KOR	0.0093	0.2657	0.6759	0.0306	0.0092	0.0092
TOG	0.0092	0.0136	0.0093	0.9494	0.0092	0.0092
OTR	0.0095	0.0171	0.0097	0.2779	0.6763	0.0094
POC	0.0083	0.0088	0.0082	0.2912	0.0084	0.6751
