# Supplementary material for: Connectivity Among Populations of the Top Shell Gibbula divaricata in the Adriatic Sea
Source: Front Genet. 2019 Mar 8;10:177. doi: 10.3389/fgene.2019.00177 (PMC6418013; doi:10.3389/fgene.2019.00177)
Supplement: Supplementary file 6 [file Table_6.pdf]

| Population | Origin | | | | | | | | |
|---|---|---|---|---|---|---|---|---|---|
| | **Self** | **KAP** | **BOK** | **KOR** | **TOG** | **OTR** | **POC** | **Unknown** | **Total** |
| **KAP** | 14 | - | - | - | - | - | 1 | 1 | 2 |
| **BOK** | 29 | - | - | 2 | - | - | - | 4 | 6 |
| **KOR** | 22 | 1 | 1 | - | - | - | 1 | 5 | 8 |
| **TOG** | 27 | - | 1 | - | - | 1 | 1 | - | 3 |
| **OTR** | 23 | - | 1 | 2 | - | - | 3 | - | 6 |
| **POC** | 29 | - | - | - | - | - | - | 5 | 5 |
| **Total** | 144 (82.7%) | 1 | 3 | 4 | 0 | 1 | 6 | 15 | 30(17.2%) |
