# Supplementary material for: Connectivity Among Populations of the Top Shell Gibbula divaricata in the Adriatic Sea
Source: Front Genet. 2019 Mar 8;10:177. doi: 10.3389/fgene.2019.00177 (PMC6418013; doi:10.3389/fgene.2019.00177)
Supplement: Supplementary file 7 [file Data_Sheet_1.docx]

**FigureS1**

Upper; Mean log probability of the data for 20 runs at each *K* (10). Bars indicate standard deviations. Lower; rate of change in the log probability between *K* values. Bottom; Evvano table values. Left; using “Popinfo” function. Right; using “Popinfo” + “location prior” functions.

**TableS1**

Effective population sizes (*Ne*) for the six populations of *G. divaricata* in the central-south Adriatic Sea. *Ne* were estimated in NeEstimator v2.1 (Do *et al.*, 2014) by three different methods (linkage disequilibrium, heterozygote excess, and molecular coancestry), together with their confidence intervals (either by parametric chi-square approximation or jackknifing over individuals). Rare alleles, which may bias *Ne* estimates, were excluded if they were found below a particular critical frequency (PCrit.). We estimated *Ne* at three different PCrit. values (0.05; 0.02; 0.01).

**TableS2**

Genetic differentiation among populations of *G. divaricata*. We estimated the statistic ß per population and their corresponding confidence intervals (95%) with the function ‘betas’ in the R package hiersfstat (Goudet, 2004).

**TableS3**

Mutation-scaled migration rates (M) among populations of *G. divaricata*, as estimated in Migrate-n v3.6.11 (Beerli & Palczewski, 2010).

**TableS4**

Ranking of the six models according to their probability, calculated with log Bayes factors in mtraceR (Pacioni *et al.*, 2015). Panmixia [a single population (KAP+BOK+KOR+TOG+OTR+POC)], two-pops [Adriatic (KAP+BOK+KOR) vs. Apulian (TOG+OTR) + Ionian (POC) sides], three-pops[Adriatic (KAP+BOK+KOR) vs. Apulian (TOG+OTR) vs. Ionian (POC) sides], six-pops (six populations), asymmetric_1 (favouring counter-clockwise dispersal following the sea surface currents in the Adriatic) and asymmetric_2 (against sea surface currents).

**TableS5**

BayesAss migration rates between populations with the following parameters: number of iterations, 10,000,000; sampling frequency, 100; length of burn-in 1,000,000; delta allele, 0.1; delta migration, 0.2; and delta *F*, 0.1.

**TableS6**

Assignment test of *G. divaricata* in the six Adriatic and Ionian populations based on 21 microsatellites. For each site (acronyms as in Table 1), individuals are presented in rows according to their sampling site and classified as individuals either assigned to their own population (Self) or to other sites or an unknown population. The last column lists the total number and percentage of individuals that were not assigned to the population from which they were sampled.
