# Supplementary figures and images for: Connectivity Among Populations of the Top Shell Gibbula divaricata in the Adriatic Sea
Source: Front Genet. 2019 Mar 8;10:177. doi: 10.3389/fgene.2019.00177 (PMC6418013; doi:10.3389/fgene.2019.00177)

L(K)

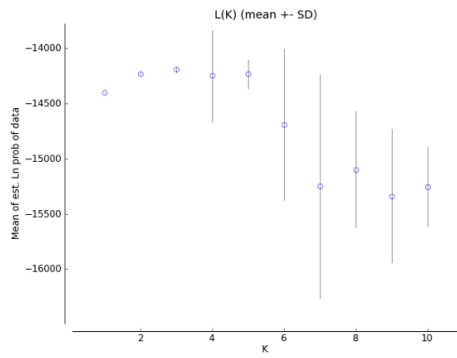

L(K)

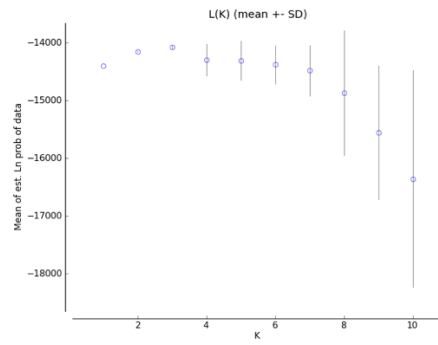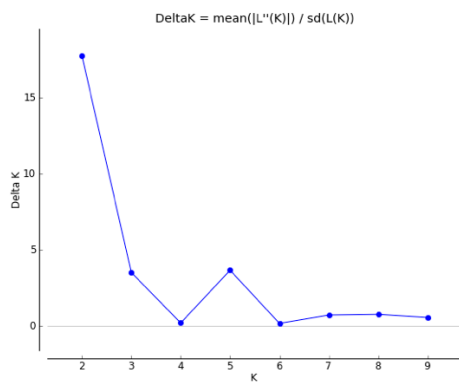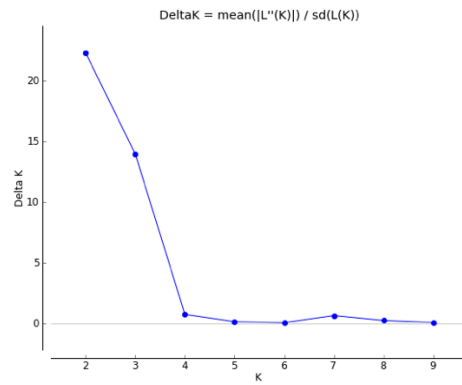

| K  | Reps | Mean LnP(K)   | Stdev LnP(K) | Ln'(K)      | Ln''(K)    | Delta K   |
|----|------|---------------|--------------|-------------|------------|-----------|
| 1  | 20   | -14403.860000 | 1.169975     | —           | —          | —         |
| 2  | 20   | -14228.745000 | 7.781522     | 175.115000  | 138.300000 | 17.772874 |
| 3  | 20   | -14191.930000 | 26.486881    | 36.815000   | 92.505000  | 3.492484  |
| 4  | 20   | -14247.620000 | 414.659194   | -55.690000  | 70.995000  | 0.171213  |
| 5  | 20   | -14232.315000 | 130.187526   | 15.305000   | 474.405000 | 3.644013  |
| 6  | 20   | -14691.415000 | 685.671140   | -459.100000 | 96.910000  | 0.141336  |
| 7  | 20   | -15247.425000 | 1017.340189  | -556.010000 | 704.355000 | 0.692350  |
| 8  | 20   | -15099.080000 | 525.358231   | 148.345000  | 387.195000 | 0.737011  |
| 9  | 20   | -15337.930000 | 602.625671   | -238.850000 | 319.670000 | 0.530462  |
| 10 | 20   | -15257.110000 | 360.222414   | 80.820000   | —          | —         |

| K  | Reps | Mean LnP(K)   | Stdev LnP(K) | Ln'(K)      | Ln''(K)    | Delta K   |
|----|------|---------------|--------------|-------------|------------|-----------|
| 1  | 20   | -14404.510000 | 0.858947     | —           | —          | —         |
| 2  | 20   | -14151.155000 | 8.159236     | 253.355000  | 181.840000 | 22.286400 |
| 3  | 20   | -14079.640000 | 20.850733    | 71.515000   | 291.145000 | 13.963298 |
| 4  | 20   | -14299.270000 | 267.387478   | -219.630000 | 205.490000 | 0.768510  |
| 5  | 20   | -14313.410000 | 335.926861   | -14.140000  | 56.735000  | 0.168891  |
| 6  | 20   | -14384.285000 | 324.720479   | -70.875000  | 28.345000  | 0.087290  |
| 7  | 20   | -14483.505000 | 435.897365   | -99.220000  | 292.790000 | 0.671695  |
| 8  | 20   | -14875.515000 | 1077.618704  | -392.010000 | 289.730000 | 0.268861  |
| 9  | 20   | -15557.255000 | 1151.184820  | -681.740000 | 119.905000 | 0.104158  |
| 10 | 20   | -16358.900000 | 1871.600382  | -801.645000 | —          | —         |

Supplement: Supplementary file 8 [file Image_1.pdf]
